# Technology Is out There for the Betterment of Us: African American Family Caregivers and COVID-19

**DOI:** 10.1093/geroni/igab046.1803

**Published:** 2021-12-17

**Authors:** Afeez Hazzan, Carol D'Agostino, Phyllis Jackson

**Affiliations:** 1 State University of New York at Brockport, Brockport, New York, United States; 2 Geriatric Mental Health Specialist, Geriatric Mental Health Specialist, New York, United States; 3 Common Ground Health, Common Ground Health, New York, United States

## Abstract

Unpaid family caregivers are mostly responsible for bearing the costs associated with caring for older adults with dementia. Importantly, the ongoing COVID-19 pandemic has created unforeseen challenges for many family caregivers. Specifically, the restrictions put in place to limit the spread of the coronavirus may be exacerbating the challenges faced by these caregivers as they try to navigate the system. Further, studies have shown that family caregivers who are members of a racial or ethnic minority group such as African-Americans or Hispanics face unique challenges when caring for their loved ones. Additional challenges may include socioeconomic disadvantages, health disparities, and language barriers that make it more difficult to access healthcare and social services. In this study, we examined the perspectives of African-American family caregivers of older adults on the feasibility of utilizing technology as a coping strategy (including for research participation) during the ongoing COVID-19 pandemic. The research question was: What are the perspectives of African-American family caregivers of people with dementia on the feasibility, opportunities, and challenges of technology as a means to engage family caregivers during a pandemic? In-depth one-on-one interviews were conducted with 12 African-American/black family caregivers. Thematic analysis of the qualitative data yielded the following three themes: (1) Acceptance that technology will play a greater role in the world going forward, and family caregivers need to adapt; (2) Opportunities to avoid social isolation while maintaining links with critical community resources; and (3) Challenges due to possible loss of privacy and lack of physical interactions

